# Whole-genome re-sequencing data to infer historical demography and speciation processes in land snails: the study of two *Candidula* sister species

**DOI:** 10.1098/rstb.2020.0156

**Published:** 2021-05-24

**Authors:** Luis J. Chueca, Tilman Schell, Markus Pfenninger

**Affiliations:** ^1^ LOEWE-Centre for Translational Biodiversity Genomics (LOEWE-TBG), Senckenberg Nature Research Society, 60325 Frankfurt am Main, Germany; ^2^ Department of Zoology and Animal Cell Biology, University of the Basque Country (UPV-EHU), 01006 Vitoria-Gasteiz, Spain; ^3^ Molecular Ecology, Senckenberg Biodiversity and Climate Research Centre, Senckenberganlage 25, 60325 Frankfurt am Main, Germany; ^4^ Institute of Organismic and Molecular Evolution (iOME), Faculty of Biology, Johannes Gutenberg University, 55128 Mainz, Germany

**Keywords:** approximate Bayesian computation, demographic history, ecological speciation, Gastropoda, gene flow, whole-genome re-sequencing

## Abstract

Despite the global biodiversity of terrestrial gastropods and their ecological and economic importance, the genomic basis of ecological adaptation and speciation in land snail taxa is still largely unknown. Here, we combined whole-genome re-sequencing with population genomics to evaluate the historical demography and the speciation process of two closely related species of land snails from western Europe, *Candidula unifasciata* and *C. rugosiuscula*. Historical demographic analysis indicated fluctuations in the size of ancestral populations, probably driven by Pleistocene climatic fluctuations. Although the current population distributions of both species do not overlap, our approximate Bayesian computation model selection approach on several speciation scenarios suggested that gene flow has occurred throughout the divergence process until recently. Positively selected genes diverging early in the process were associated with intragenomic and cyto-nuclear incompatibilities, respectively, potentially fostering reproductive isolation as well as ecological divergence. Our results suggested that the speciation between species entails complex processes involving both gene flow and ecological speciation, and that further research based on whole-genome data can provide valuable understanding on species divergence.

This article is part of the Theo Murphy meeting issue ‘Molluscan genomics: broad insights and future directions for a neglected phylum’.

## 1. Introduction

Unravelling how species diverge on the genomic level has been a central theme in evolutionary biology during the past years. Although allopatric speciation, implying divergence in the absence of gene flow, is the most widely accepted mechanism for the origin of species diversity, speciation with gene flow is now well documented [1–4]. Even well-defined species can coexist in a long-term stable selection–migration–drift equilibrium [5,6]. However, continuing gene flow among diverging species is also influenced by the complex interplay between geography, ecology and selection [7]. This makes the inference of geographical divergence scenarios in the past difficult. Recent advances in sequencing technologies are offering exciting new and revolutionary insights into genomic differentiation patterns between recently diverged species [8–10]. Such ‘palaeogenomics’ studies [11] are, therefore, suitable to unravel the speciation history of current biodiversity. Unfortunately, genomic speciation studies continue to show a strong bias to model organisms and vertebrates [12], while studies on the most diverse groups like invertebrates in general and molluscs, in particular, are still scarce.

Owing to their low dispersal ability and strong population structure, land snails have been commonly used during the past two decades in phylogeography, molecular phylogenetics and evolutionary studies [13–16]. However, the lack of reference genomes has up to now prevented more comprehensive studies on their speciation history and molecular evolution.

**Figure 1.**
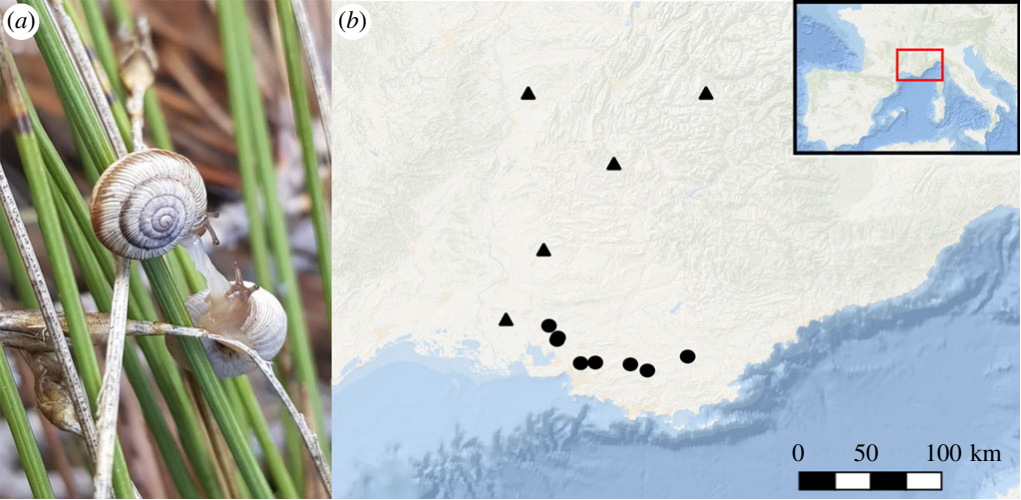
(*a*) Two specimens of *C. rugosiuscula* mating (Pélissanne, Provence–Alpes–Côte d'Azur, France. Copyright © L.J. Chueca). (*b*) *Candidula* sampling locations for this study (for the sake of clarity, the population from Germany is not shown). Triangles correspond to *C. unifasciata* populations, whereas dots indicate *C. rugosiuscula* populations. See electronic supplementary material, table S1, for further details on data collection.

In addition to genomic resources, speciation studies require in-depth knowledge on distribution, ecology and population history of the diverging species [17]. *Candidula unifasciata* and *C. rugosiuscula* are two closely related land snail species from the Western Palearctic [18,19], for which this information is available. The species are small (5–10 mm), with a whitish shell that may be banded. Both species occur in open and dry areas with sparse vegetation on the calcareous underground. While *C*. *unifasciata* is widespread from southeast France and Italy to central Europe [19,20]; *C*. *rugosiuscula* is restricted to the southern Provence region in France [18] (figure 1). The two species have distinct climatic niches, which, together with associated, known shell adaptations, led to the hypothesis of an ecological speciation [20]. The climatic fluctuations of the Pleistocene caused substantial range (and probably demographic) dynamics in the two species [14,20,21], as (sub)fossil and phylogeographical evidence showed. As for *C. unifasciata*, previous studies have suggested that its niche seems to have partially evolved after the last glacial maximum (LGM) to fit the re-emerging Mediterranean climate, while other populations have tracked their ancestral niche in a range expansion to the North [14]. Yet, there has been no evidence of niche evolution in the case of *C. rugosiuscula*. Currently, although there is no obvious geographical barrier, their distributions' ranges do not overlap and no co-occurrence locality is known. However, given the range dynamics in the past, we hypothesize that this could have been different during their history [22], which makes these two sister species an ideal study case to delve into the genomic underpinnings of speciation processes.

In this study, we combined the recently sequenced reference genome of *C. unifasciata* with individual re-sequencing data from the two closely related species in order to achieve two main goals. First, we aimed to infer the demographic history of both taxa by means of coalescent simulation analyses. Second, we explored the temporal divergence of differentially selected genes, to provide a better understanding of the potential cause of the initial divergence between species.

## 2. Material and methods

### (a) Taxon sampling, DNA isolation and sequencing

All specimens were collected between 2002 and 2013 from 14 populations, mainly from the potential contact zone between the two species (figure 1*b*) and preserved in absolute ethanol (see electronic supplementary material, table S1). Total genomic DNA was extracted from foot muscle of specimens using DNeasy Blood and Tissue Kit (Qiagen, USA), and stored in double-distilled water. Specimens were photographed and DNA barcoded with 16S and COI (see electronic supplementary material) to assign them to the target species *C. rugosiuscula* and *C. unifasciata*, respectively. Whole-genome re-sequencing was carried out on an Illumina NovaSeq 6000 platform, by Novogene Company (Beijing, China), to generate 150 bp paired-end reads per sample. All samples were sequenced to a target coverage of 15X. Raw data have been deposited at European Nucleotide Archive (ENA) BioProject number: PRJEB41103. Quality trimming was performed with Trimmomatic v. 0.36 [23] by using the wrapper autotrim v. 0.6.1 [24] (see electronic supplementary material for further details). Final overall quality was checked using FastQC v. 0.11.8 [25] and summarized with MultiQC v. 1.9 [26].

### (b) Variant calling and filtering

All raw sequence reads were mapped against a repeat-masked *C. unifasciata* genome (ENA: PRJEB41346; GCA_905116865 [27]) with backmap.pl (https://github.com/schellt/backmap) in combination with BWA mem v. 0.7.17 [28], SAMtools 1.10 [29], Qualimap v. 2.2.1 [30] and MultiQC. PCR duplicates were identified and filtered with MarkDuplicatesSpark from GATK v. 4.1.7 [31,32]. After that, variants were called using GATK HaplotypeCaller in GVCF mode. Then, we applied a hard-filtering by running GATK VariantFiltration and SelectVariants, where indels were filtered out. In addition, by using VCFtools v. 0.1.17 [33], we excluded sites with a tolerance of 10% for missing data by simultaneously applying the following filters: mean number of reads per individual smaller than 10 or larger than 50, genotype quality smaller than 30, minimum allele frequency less than 0.1.

### (c) Population structure analyses

Genetic admixture between target species was estimated using ADMIXTURE v. 1.3.0 [34]. The VCF file was converted to PLINK's PED format using VCFtools v. 0.1.17 [33] and PLINK v. 1.9 [35,36] with parameter-indep-pairwise 50 10 0.1 to reduce the linkage disequilibrium effect. Log-likelihood values for an increasing number (*K*), from *K* = 1 to *K* = 10, were estimated and 200 bootstrap replicates were used to calculate cross-validation errors. The optimal *K* was indicated by the lowest cross-validation error. In addition, a principal component analysis (PCA) was conducted on unlinked single-nucleotide polymorphisms (SNPs) using the R package *factoextra* v. 1.0.7 [37].

To test the relative levels of gene flow between both species, the proportion of rare SNPs shared between them was calculated [38]. We considered SNPs with frequencies from 2 to 5 (allele frequencies between 4% and 10%) as rare alleles. Because rare alleles are expected, on average, to have been originated only recently, they will be limited to a single population or species if recent gene flow is absent [39].

### (d) Historical demography

PSMC [40] was employed on consensus genomic sequence data to characterize historical demography by examining heterozygosity densities. We applied PSMC with input files generated using SAMtools mpileup v. 1.9 [29] and by applying a minimum mapping and base quality of 30. PSMC was run with 20 iterations and the upper limit of TMRCA was set to 20, initial *ρ*/*θ* value to 5, and *N_e_* was inferred across 100 interval times (8 + 40 × 2 + 6 + 6). Results were scaled with a plausible but not empirically derived mutation rate of *µ* = 1 × 10^−8^ per base pair and generation [41], assuming a generation time of 1 year.

### (e) Demographic history scenarios and inferences by approximate Bayesian computation

We tested five demographic models simulating plausible divergence scenarios, considering temporally different gene flow between the two *Candidula* species. To do so, we took into account the PSMC results with regard to divergence time and demographical trajectory. Each model assumed a divergence around 1 Ma (*T*_split_) of the ancestral population size *N*_ANC_ into two species *N*_1_ (*C. unifasciata*) and *N*_2_ (*C. rugosiuscula*). The complete isolation scenario (Model 1) assumed that divergence occurred without post-speciation gene flow between both species. The other four models differed by the temporal pattern of interspecies gene flow. Model 2 corresponds to a gradual scenario resulting in complete isolation relatively soon after divergence (*ca* 200 kya), Model 3 to secondary contact during the last glaciation phase (100–10 kya), Model 4 to secondary contact during the last warm phase (150–100 kya) and Model 5 to constant gene flow until recently (10 kya).

We conducted coalescent simulations of the multidimensional site-frequency spectrum (SFS) as summary statistics. To minimize the effects of selection on demographic inference, only presumably neutrally evolving SNPs at fourfold degenerated sites were analysed [38]. We randomly selected six individuals per species and obtained all fourfold degenerated SNP sites in the genome by using tbg-tools v. 0.2 (https://github.com/Croxa/tbg-tools). The selected SNPs were pruned for linkage disequilibrium with PLINK, applying an *r*^2^ threshold of 0.1. The unlinked, neutral SNPs were used to obtain the observed SFS with easySFS (https://github.com/isaacovercast/easySFS). We ran 100 000 simulations with fastsimcoal2 v. 2.6.0.3 [42] for each model, based on the observed SFS. The simulated SFS obtained for each model were then compared in an approximate Bayesian computation (ABC) framework [43] to the observed SFS.

### (f) Estimation of genetic diversity

We estimated four metrics representing different measures of population differentiation. We calculated absolute divergence (*D*_XY_) with popgenWindows.py (https://github.com/simonhmartin/genomics_general/blob/master/popgenWindows.py) [44]. In addition, we assessed the fixation index (*F*_ST_ [45], Tajima's *D* [46] and nucleotide diversity *π* [47] in 30 kb windows using VCFtools.

We estimated differences between species for Tajima's *D* (TD) and genetic diversity (*π*) by computing Mann–Whitney *U* statistical tests using the R package ggstatsplot v. 0.6.1 [48].

### (g) Early diverged windows

To obtain information about how *Candidula* genome landscape has evolved, we assumed that windows with higher net divergence ancestral diversity (*D*_XY_) tend to have diverged at earlier stages [49]. We sorted all 30 kb non-overlapping windows by their *D*_XY_ value and defined those regions expected to have diverged at earlier stages (*W*_early_) as those above a value of 0.35.

### (h) Selection on protein-coding genes

To relate adaptive protein evolution to the divergence process, we selected the coding sequence (CDS) from all genes present in the *C. unifasciata* genome annotation. We calculated the McDonald–Kreitman test (MKT) by running PopGenome v. 2.2.4 [50] on all samples, with a Fisher's exact significance test. For significant genes, the neutrality index and the proportion of divergent SNPs fixed by positive selection (*α*, [51]) were calculated.

Putative gene functions were obtained from the UniProt website (https://www.uniprot.org; accessed 3.11.2020). We searched for connections between the identified genes and divergence processes by literature research in Google Scholar, using the gene name (together with its associated pathway) and *speciation* as search terms. We conducted gene ontology (GO) term enrichment analyses on the category Biological Process on all genes that were present within the early diverged windows, using all 13 221 GO-annotated genes as the so-called *universe* parameter. The analysis was carried out using the R package *topGO* v. 2.42 [52]. Only terms with more than 5 annotated genes were considered.

## 3. Results

We generated whole-genome re-sequencing data for 10 *C. unifasciata* and 14 *C. rugosiuscula* individuals. The final mean coverage of uniquely mapped reads per site was 16.9X and 17.3X in samples of *C. unifasciata* and *C. rugosiuscula*, respectively. A total of 11 887 421 SNPs were found in the 24 *Candidula* individuals.

### (a) Population structure

**Figure 2.**
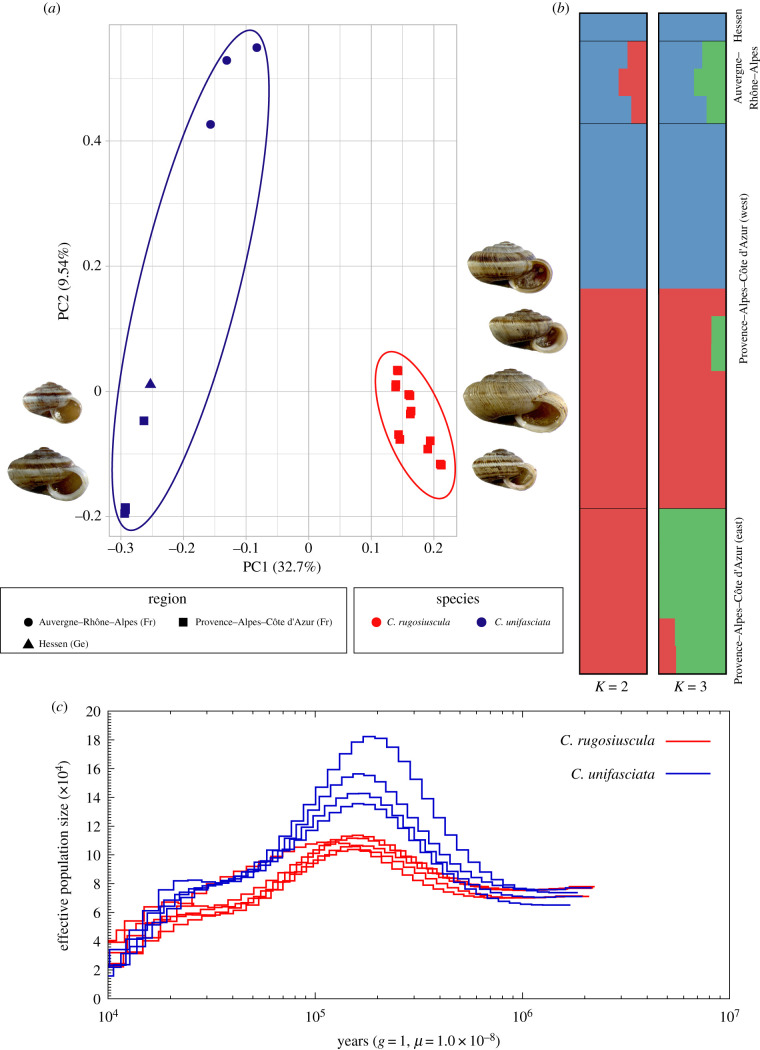
(*a*) Principal component analyses (PCA) corresponding to the LD-pruned high-quality SNPs set. (*b*) Population structuring plots based on ADMIXTURE analysis with *K* = 2 and *K* = 3. The *x*-axes quantify the proportion of an individual's variation from inferred ancestral populations; the *y*-axes show the different individuals of *C. rugosiuscula* and *C. unifasciata* populations. (*c*) Historical effective population size (*N*_e_) obtained by PSMC analysis for selected *Candidula* specimens. In all plots C. *rugosiuscula* and *C. unifasciata* are represented in red and blue, respectively.

Both clustering methods (PCA and ADMIXTURE) found high statistical support for two clusters. The PCA joined two species-specific clusters according to taxonomic assignment (figure 2*a*), with the first two components explaining 32.7% (PC1) and 9.5% (PC2) of the total variance. PC1 separates *C. unifasciata* from *C. rugosiuscula*, whereas PC2 reflected the intraspecific divergence of *C. unifasciata* with two groups (Hessen + Provence and Auvergne–Rhône–Alpes). ADMIXTURE further showed that the best scenario recovered two genetic groups (*K* = 2, highest log-likelihood value = −221 856 262.7 and lowest CV-error = 0.72). Under the *K* = 3 scenario, two well-differentiated clusters were recovered within *C. rugosiuscula*, grouping the eastern and western populations (figure 2*b*).

### (b) Historical demography

The temporal trajectory of the effective population size (*N*_e_) for both *Candidula* species is shown in figure 2*c*. Both species recovered similar demographic histories, which showed significant population growth from beginning of the middle Pleistocene (*ca* 800 kya) until reaching the *N*_e_ peak approximately at 110 kya. The trajectories diverged about 1 Ma, indicating the timing of species split. However, the growth rate of *C. unifasciata* was higher than that of *C. rugosiuscula*. The effective population size of both species decreased until minimum levels at the beginning of the Holocene.

### (c) Scenarios of speciation

**Figure 3.**
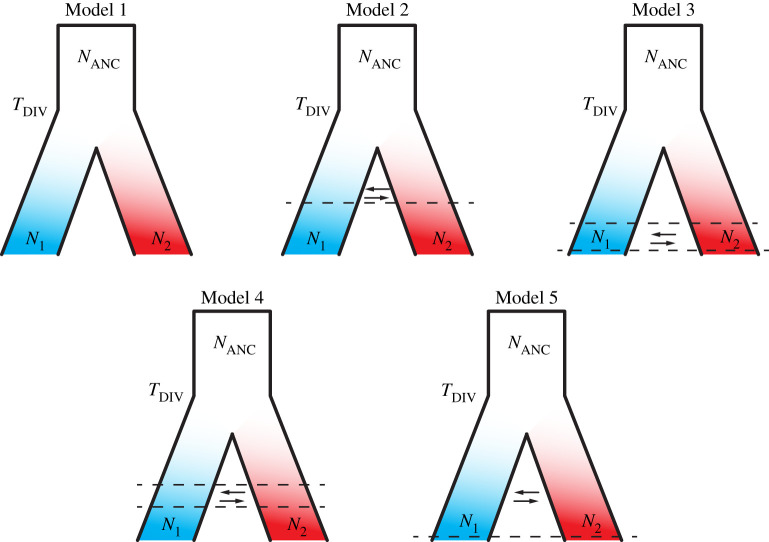
Demographic history scenarios tested in this study, which included parameters for divergence time (*T*_DIV_), ancestral population sizes (*N*_ANC_), *C. unifasciata* and *C. rugosiuscula* population sizes (*N*_1_ and *N*_2_, respectively) and gene flow (arrows) when applicable. Model 1: divergence in complete isolation; Model 2: divergence with gene flow until *ca* 200 kya; Model 3: divergence with gene flow from 100 to 10 kya; Model 4: divergence with gene flow between 150 and 100 kya; and Model 5: divergence uninterrupted until 10 kya. The proportions of accepted simulations by ABC analysis were: M5 (42.9%) > M3 (23.9%) > M2 (19.0%) > M4 = M1 (7.1%). (Online version in colour.)

All five tested models recovered a proportion of accepted simulations, highlighting the complexity to select the best scenario. Scenarios including at least some post-speciation gene flow in total covered 92.9% of all accepted simulations. Nevertheless, Model 5 (M5), which indicated gene flow since the divergence time until approximately 10 kya, recovered the highest proportion of simulations, 42.9% (figure 3). The rest of the other models showing gene flow between species (i.e. M2, M3 and M4) also recovered an important proportion of accepted simulations: 19.0%, 23.9% and 7.1%, respectively. Finally, the only scenario without gene flow (Model 1), recovered the remaining 7.1% of the accepted simulations.

### (d) Genetic differentiation

The absolute divergence at the interspecific level (*D*_XY_) was much higher than mean genetic diversity at the intraspecific level (*π*) (*D*_XY_ = 0.2507 ± 0.0342 compared to *π_C. unifasciata_* = 0.0029 ± 0.0018 and to *π_C. rugosiuscula_* = 0.0037 ± 0.0017).

**Figure 4.**
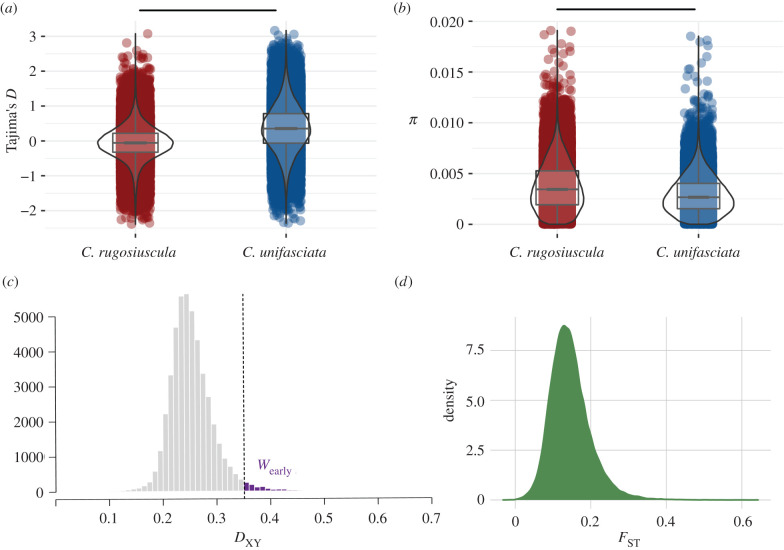
Relation of population genetic statistics in 30 kb sliding windows. (*a*) Violin plots representing the distribution of Tajima's *D* for each of the species under study. (*b*) Violin plots representing the distribution of genetic diversity (π) for each of the species and window group under study. (*c*) Histogram representing the distribution of absolute divergence values (*D*_XY_). Values corresponding to those regions expected to have diverged earlier are shown in purple (*W*_early_). (*d*) Density plot representing the distribution of the fixation index (*F*_ST_). Note that in (*a*) and (*b*) red colours (left) correspond to *C. rugosiuscula*, whereas blue colours (right) represent the distributions for *C. unifasciata*. Asterisks indicate significant differences between species based on Mann–Whitney *U* tests (*p-value < 0.001).

Mean TD exhibited different values between species (figure 4*a*). In particular, mean TD for *C. rugosisucula* was close to zero, while mean TD was positive and significantly higher for *C. unifasciata* than for *C. rugosiuscula*. (Mann–Whitney *U* = 20.26, *p* < 0.001).

Differences in genetic variation (*π*) were also found between the two species, being significantly smaller for *C. unifasciata* than for *C. rugosiuscula* (figure 4*b*, Mann–Whitney *U* = 20.27, *p* < 0.001).

### (e) Selection on protein-coding genes

From the total 22 464 genes annotated in the *C. unifasciata* genome, the MKT showed a significant accumulation of either synonymous or non-synonymous divergent SNPs for 133 of them. Neutrality index indicated that 73 of these genes were under negative selection, whereas 60 showed signs of positive selection. Besides, 16 of these 60 positively selected genes were identified within the early diverged windows (*W*_early_) (see electronic supplementary material, table S2).

Of the 60 positively selected genes, 51 could be annotated. Of these, 10 were genes relevant for important intragenomic molecular interactions, eight associated with cyto-nuclear compatibility and 10 with ecological adaptations. Six genes are involved in gene transcription regulation, which could not be linked with particular biological processes (see electronic supplementary material, table S2). A GO analysis revealed that after the false discovery rate (FDR) correction, no GO term was significantly enriched among the genes in the regions diverging early in the process (*W*_early_) (electronic supplementary material, table S3).

## 4. Discussion

Here, we used, for the first time, a population genomic approach to investigate the speciation history of two closely related land snail species, *C. unifasciata* and *C. rugosiuscula*. Our analyses, based on individual whole-genome re-sequencing data, showed that the two species are high genetically divergent. Overall, our results further suggest that divergence between these species might have been a complex process involving both post-speciation gene flow and ecological speciation.

### (a) Historical demography of speciation

Results from the genome-wide clustering analyses confirmed that the two studied taxa are overall highly genetically differentiated, in accordance with previous phylogenetic studies [19] and mitochondrial assessments [18]. These genome-wide results thus justified the diagnosis of two different species, recovering the valid species status of the taxon *C. rugosiuscula* [18]. Despite their substantial divergence, a few *C. unifasciata* individuals from Auvergne–Rhônes–Alpes populations showed signs of some relatively recent admixture with *C. rugosiuscula*. Such recent introgressive gene flow between the divergent taxa should manifest in sharing a proportion of rare SNPs [38]. Indeed, about 4% of rare SNPs were shared between these *C. unifasciata* populations and *C. rugosiuscula*. This indicated that reproductive isolation between the two taxa is not yet complete. Nevertheless, this apparently recent admixture requires an explanation, because both species are currently allopatrically distributed and the closest known *C. rugosiuscula* populations are quite distant from the introgressed population [22]. While land snails are proverbially poor active dispersers, vertebrates may serve as long-distance dispersal vectors of snails [53–55]. Long-range passive dispersal along traditional sheep trails in southern France was inferred previously as the most likely cause for admixture between divergent *C. unifasciata* populations [14] and is the most likely explanation here as well [56].

The effective population sizes of both species have experienced major changes since their split, most likely as a result of the important climatic fluctuations occurring in western Europe during the Pleistocene. Overall, the estimated population size for *C. unifasciata* was always higher than that of *C. rugosiuscula*. Maximum effective population size in both species was reached during the last interglacial period (the Eemian, *ca* 129–116 kya) when the environmental conditions were warmer and wetter [57–59]. Moreover, this period was characterized in Europe by a pronounced rise of grasslands [60,61], the most suitable habitat for *Candidula* species, potentially allowing their expansion. After that, the global cooling corresponding to the Last Glacial Period (*ca* 115–12 kya) could have caused the observed decline of both *Candidula* populations until the Holocene. All time estimates depend, however, on the mutation rate applied, for which no empirical estimate exists. Nevertheless, the obtained divergence time estimate concurs with previous mitochondrial estimates [22]. The Holocene population expansion of *C. unifasciata* inferred previously by phylogeographical methods [20] could not be resolved by PMSC with its limited power to infer very recent events [62].

These population histories were also reflected in the distributions of TD. For both species, only very few windows were outside the range of −2 to +2, which is usually assumed as evolving neutrally. While the means of the distributions for the early and late diverged windows in *C. rugosiuscula* are both close to zero, the means of *C. unifasciata* for both periods are shifted to the positive side, probably reflecting genome-wide effects of the population expansions during the last interglacial period (figure 2*c*) and after the LGM [20].

### (b) Speciation with gene flow

ABC methods allow the testing of customized, complex demographic models by comparing simulated data with empirically observed data [63,64]. Nevertheless, ABC approaches can only distinguish between the tested scenarios, which may or may not reflect reality [65]. In the past years, several studies have successfully used this approach by employing the SFS as summary statistics to infer the demographic histories of non-model organisms [64,66–68]. We decided to test models of divergence that consider each of the two diverging species as an unstructured population. Given the known strong population structure of land snails in general [69] and *Candidula* in particular [14], this is an oversimplification. However, this likely did not influence the inferences from SFS because we used individuals from several populations [70]. The vast majority of the accepted coalescent simulations (92.7%) came from models with post-speciation gene flow. The best-supported model suggested a divergence with at least occasional gene flow until 10 000 years ago [71,72].

Divergence with gene flow is a progressive process, initiated by selection targeting local genomic regions and expanding from there over time, eventually leading to genomic and reproductive isolation [73,74]. The fact that the divergence far exceeded intraspecific diversity suggests that the genomic isolation among the sister species is largely complete. Under speciation with gene flow, the distribution of interspecific divergence values should contain information about the temporal trajectory of the divergence process, because the different parts of the genome can start to accumulate divergent mutations only if these parts are isolated from each other by selection [5]. The left-skewed shape of the net divergence distribution *D*_XY_ with its long tail suggests that the divergence process started with small parts of the genome, accelerated at some point, to slow down finally after most of the genome became isolated.

### (c) Processes driving divergence

The MKT showed that genes associated with several known speciation processes were positively selected among the closely related taxa. These included genes fitting to the initial ecological speciation hypothesis, but genes prone to molecular and sexual incompatibility were also detected (electronic supplementary material, table S2). Because the MKT test identifies only selected genes with several amino acid changes, genes diverged in their transcription regulation were not recorded. The positively selected genes were functionally associated with cyto-nuclear and histone incompatibilities, spermatogenesis, gamete recognition or sex development, all of which were already invoked in speciation of snails [75–79]. In addition, we also identified genes likely involved in ecological adaptation. They were responsible for functions like thermal tolerance, dietary switch, biomineralisation of the shell and pigmentation. These functions fit well to the observed niche differences between the species. *C. rugosiuscula* occurs in a warmer climate than *C. unifasciata*, which is associated with a markedly different vegetation, the basis of the snail's diet [22]. In addition, the shells of the species are markedly different in response to the different climate [18], a response well known for snails [80].

Among the genes that likely diverged first and could thus potentially have given some insight on processes that initiated the divergence, none of the above processes prevailed (see electronic supplementary material, table S2). There was also no GO term significantly enriched among the genes in the regions diverging early in the process. With the data at hand, it is, therefore, not possible to support or reject the hypothesis of an initially ecological speciation. The current analysis, however, yielded substantial insight into the temporal demographic trajectory of the speciation and suggests that both genomic and ecological processes might have played a role in this divergence.

## 5. Conclusion

Here, we provide insights into the evolutionary histories and the mechanisms of speciation for two closely related land snail species, *C. unifasciata* and *C. rugosiuscula*. Namely, we identified long periods of gene flow during the speciation, where the main genes involved were associated with reproductive incompatibility and ecological functions. We foresee that a better integration of whole-genome re-sequencing data on evolutionary ecology studies will improve our mechanistic understanding on how species diverge. Future studies could expand our results by considering other species to test the generality of our findings and to further investigate the complex and challenging speciation process in land snails.

## Data accessibility

Whole-genome individual re-sequencing raw data are available at European Nucleotide Archive (ENA) BioProject number: PRJEB41103.
